# Assessment of logistics service quality dimensions: a qualitative approach

**DOI:** 10.1186/s41072-021-00095-1

**Published:** 2021-09-22

**Authors:** Gamze Arabelen, Hasan Tolga Kaya

**Affiliations:** 1 Maritime Faculty Department of Maritime Business Administration Management, Dokuz Eylül University, Izmir, Turkey; 2 Dokuz Eylül University, Izmir, Turkey

**Keywords:** Logistics, Service quality, Logistics service quality, Systematic literature

## Abstract

Globalization and complex supply chain networks have been affecting Logistics Services Providers’ (LSPs) service delivery and service expectations. Logistics Service Quality (LSQ) is becoming a more important aspect for LSPs and logistics service customers. In recent years, there has been an increase in the studies on service quality in logistics. Researchers have been trying to identify aspects of LSQ and its dimensions in order to create a measurement model that could be used in overall logistics services. However, there is still neither a unified nor agreed LSQ measurement model in the literature and researchers have been debating continuously on the proposed models. This paper targets to investigate and suggest LSQ measurement dimensions obtained from previous studies by analyzing the findings within a systematic approach and improving the findings with semi-structured interviews. In this study, systematic literature analysis has been conducted to research papers published in selected academic databases with specific keyword and keyword cluster searches to identify the related articles published within a specified period. Papers have been selected in accordance with the predefined criteria. As a result, a total of 59 articles have been determined for the search criteria and the findings obtained were analyzed. Most frequently used research trends and methods on service quality in logistics have been identified. In addition, the most frequently used LSQ dimensions and factors have been reviewed. Moreover, the most frequently used service quality approaches and measurement models have been analyzed. The results received from systematic literature review have been composed and dimensions have been identified. Semi-structured interviews with LSPs and customers of LSPs in Germany-based companies have been conducted to strengthen the findings gained from systematic literature review. 5 LSQ dimensions and 24 factors have been formed with the help of semi-structured interviews. This paper represents the basis for further research for empirical studies and can be used as a guideline for quality management practices in logistics applications and transport.

## Introduction

Globalization and growing supply chain networks have been pushing logistics service providers to focus on the provided logistics practices. Simultaneously, service types offered by logistics service providers have increased quickly. Importance of logistics services also has been increased universally; hence, service quality has become an important driver for LSPs. The importance of logistics services is known by practitioners and academics. Significance and interest in Logistics service quality (LSQ) have been also increasing. The concept of LSQ is equally important for customers and LSPs (Mentzer et al. 1999; Thai 2013). High level of LSQ increases logistics providers’ competitive advantage among compelling business environments (Wang and Hu 2016). Good service quality offered to customers generates customer satisfaction as well as customer loyalty for the service provider (Franceschini and Rafele 2000; Davis and Mentzer 2006; Baki et al. 2009).

There has not been any clear understanding of the LSQ concept despite the increasing number of research papers. Major focus of the researchers has been on the concept of the LSQ and its quality attributes, how to analyze and measure the quality of the services (Bienstock et al. 1997; Mentzer et al. 1999; Franceschini and Rafele 2000; Rafele 2004). Nonetheless, researchers have developed different ideas on logistics concept and service quality dimensions over time. There have been very few studies with the holistic approach on the LSQ to analyze overall developed dimensions and the attributes along with the general framework. Therefore, a comprehensive LSQ model that would incorporate different sectors is not available at present.

General approach of the researchers developing a study in LSQ has kept the literature review part very short and directed it to particular approaches without critically viewing the literature. This paper is aiming to address the previously mentioned issue by analyzing papers related to LSQ with a systematic approach. This will ensure that previous findings from scientific papers are systematically analyzed and presented and findings can be used in future studies to develop scientific or practical LSQ studies. Additionally, this study is anticipating LSQ attributes by analyzing research trends and general usage of LSQ dimensions, research methods, and fields of sectors. Furthermore, it is aiming to conduct a semi-structured interview with logistics professionals in order to confirm and enhance the outcome of the systematic literature review.

This paper has been developed through multiple sections. In the first section, research methodology has been explained. General approach in the systematic literature review, paper selection criteria, keywords, databases, and preliminary paper classification have been described in the second section. In the third section, descriptive analysis of the selected papers has been carried out. In the fourth section, LSQ dimensions and attributes have been analyzed and the LSQ measurement model has been created to discuss the findings in semi-structured interviews. In the fourth section, semi-structured interviews and findings from business professionals’ contributions have been explained. In the fifth section, a brief overview of this study has been presented and in addition notes on future works have been provided.

## Research methodology

Systematic literature review methodology has been used in this study to have a holistic approach towards LSQ studies and interpret the findings obtained from previous papers. Systematic literature analysis method has been considered a technique of systematic, qualitative, objective, and quantitative description in the research area (Berelson 1952). A systematic content analysis methodology has been considered a very powerful and an explicit tool because of its ability to combine qualitative approaches retaining rich meaning with quantitative analyses (Duriau et al. 2007; Fink 2005). Additionally, the main difference between systematic literature review and traditional literature review has been considered the first comprehensive search section (Crossan and Apaydin 2010). In order the follow a structured method with valid results, a systematic literature review approach from the literature has been applied (Seuring and Gold 2012). In this regard, a systematic literature review has been planned in this study with several steps as: material collection, descriptive analysis, category selection, material evaluation. Material collection reflects gathering all necessary papers from previously created criteria. Collection of materials has been the most crucial step in systematic literature reviews. In the study, literature regarding the LSQ has been selected from peer-reviewed journals and literature databases, Web of Science, ScienceDirect, Emerald, Taylor and Francis, JSTOR, Business Source Premier, and the web. Second part of the systematic literature review has been descriptive analysis. Only studies in English language and published between 1995 and 2020 have been selected for the future classification. The formal characteristics of the selected papers have been set out in the descriptive analysis section to provide background for the content. Consequently, publication years, research methods and research fields of reviewed journals have been documented. Structural dimensions and related categories for future analytics have been selected in category selection. In the material evaluation section, all analyses have been presented according to determined categories and parameters.

Semi-structured interviews have been used in this study to consolidate the LSQ dimension findings from systematic literature analysis, as it is the most frequently used interview method (Taylor 2005; Dicocco-Bloom and Crabtree 2006). Flexibility and reciprocity of semi-structured interviews have benefited the LSQ discussion. Questions regarding service quality in logistics have been prepared prior to meetings, which were shaped around the systematic literature review findings and perceptions of the participants. In semi-structured interviews, following a strict structure is not advised (Kallio et al. 2016). Definite resolution on logistics quality and definition of quality dimensions have not been agreed upon for LSQ, therefore a semi-structured interview qualitative approach is considered more convenient in order to allow participants to express themselves. In order to create successful semi-structured interviews, a five-step model has been utilized (Kallio et al. 2016). Firstly, prerequisites of the interviews have been decided. Due to the coronavirus pandemic situation, related global restrictions and organizations, new working models such as online meeting method have been selected. Second step is gathering previous knowledge on data by using the systematic literature review. This has allowed the interviewer to gain knowledge and confidence in regular spontaneous follow-up questions. In the third step, guidelines of the interview have been developed. Questions have been prepared regarding participants’ understanding of LSQ, participants’ perception of the identified LSQ dimensions and follow-up questions regarding examples for the in-depth analysis of the topic. In the fourth step, a pilot has been conducted with one logistics business professional to test the clarity of the developed approach. In the final step, semi-structured interviews have been performed with five logistics professionals.

## Systematic literature review

Systematic literature review is advised to be applied to a specified period of time. Therefore, materials have been selected from research papers that were published between 1995 and 2020. Specific keywords related to service quality in logistics have been used in literature databases such as Web of Science, ScienceDirect, Emerald, Taylor and Francis, JSTOR, Business Source Premier, and the web to identify the first step. Only papers that have been peer-reviewed in English language have been selected for further analysis for systematic review. Table 1 provides a summary of sample paper selection. In literature databases with keyword matches in their titles, 221 papers that are fit for the search criteria have been found. Furthermore, the suitability of the sample has been checked by applying a two-stage screening process. First screening has been applied to the abstracts of the selected papers. After analyzing the abstracts of 221 papers, sources that were irrelevant or with little relevance to the topic have been excluded from further analysis. However, studies with no abstract or with unclear information have been directly transferred to the second stage. In the second screening process, full paper review has been applied to enforce the relevance of the selected literature sample. Additionally, papers that have been cited multiple times and fit to the criteria of this research have been included in the samples. As a result, final sample has consisted of 59 papers.

**Table 1:** Sample Selection for Systematic Literature Review

Steps	Criteria	Results
Initial sample	Samples gathered from literature databases and duplicates were removed	221
First step screening	Abstract screening of the selected papers with keyword relevance. Sources that are not related to LSQ were removed	71
Second step screening	In-depth analysis of the selected studies. Sources that had no related information about LSQ were removed	50
Additional sources for sample	Multiple cited sources in initially selected studies which fit to standards of the study	9
Final sample		59

After collecting the sample based on criteria, descriptive analysis has been followed, as it would create a framework for the systematic analysis. In this context, formal characteristics have been analyzed. Consequently, publication years and service fields have been analyzed to identify the preliminary framework of the selected literature sample. Publication years of the selected studies have shown that the trend towards the research topic of LSQ had been increasing. Findings of the study have shown that the LSQ is still a discussion subject among researchers. In order to show the academic interest in the LSQ topic, selected timeline of 25 years has been divided into five years of periods. The results have shown that 23 papers were published between 2015 and 2020, which clearly shows the increasing relevancy and interest in the research topic. Furthermore, search fields of selected papers have been analyzed and results reflected that 49% of the studies have been conducted in the logistics field and the second most popular research field groups have indicated the industrial management field with 20% of the total sample.

After analyzing the descriptive specifications of the selected research paper samples, analytic categories have been selected including research methods, data analysis methods, LSQ dimensions, service quality measurement models, approach of the studies. In the last part of the systematic literature analysis, selected categories have been analyzed and categorized to create some practical guidance on the LSQ research question. According to Avenier (2010), decontextualized evaluation of the literature analysis’ results brings out the possibility of proposing a certain degree of generalization for the findings. Therefore, systematic literature review findings have been used to identify the first design of the LSQ dimensions and later discussed in semi-structured interviews.

## Analysis of the Categories

Previously founded categories have been analyzed to create further research design with transparency. Therefore, used data analysis and research method of selected literature sample have been analyzed. Table 2 provides an overview of the used researched methodology. According to the results, linear usage of qualitative, quantitative, and multiple data analysis known as triangulation has been used among 51% of the studies and 76% of the studies have had empirical approach.

**Table 2:** Research Method of LSQ

Research approach	Publication ratio (%)
Empirical	76
Analytical	14

There is an increase in empirical studies about the LSQ topic in addition to using existing created models and trying to validate the quality measurement models. Besides, many researchers have been searching the relationship between the LSQ and other attributes such as loyalty and satisfaction. Consequently, this increase in validation studies may refer to a reaction to unconformity on the search field and in search of study and generalized LSQ measurement model. Moreover, qualitative LSQ dimensions developing studies have been mainly observed in early periods and most researchers preferred to create an LSQ model and validate its reliability by quantitative methods throughout the time. Details of the research approach method with corresponding publications has been presented in Table 3. From triangulation, Mentzer et al. (1999), created a nine-dimension service quality measurement model which is broadly used, Feng et al. (2007), Gil-Saura et al. (2008) and Thai (2013) also developed different models which were created for the need of the search in LSQ with different approaches.

**Table 3:** LSQ Research Approach Method

Method approach	Publication
Qualitative data analysis method	Caplice and Sheffi (1995), Fung and Wong (1998), Franceschini and Rafele (2000), Mentzer and Myers (2004), Rafele (2004), Davis and Mentzer (2006), Wojciechowska (2011), Rahmat and Faisol (2016), Gulc (2017)
Quantitative data analysis method	Emerson and Grimm (1996), Rutner and Langley (2000), Seth et al. (2006), Rafiq and Jaafar (2007), Gotzamani et al. (2010), Bouzaabia et al. (2013), Kavaliauskiene et al. (2014), Politis et al. (2014), Roslan et al. (2015), Lan et al. (2016), Yu et al. (2016), Kilibarda et al. (2016), Limbourg et al. (2016), Sohn et al. (2017), Murfield et al. (2017), Yumurtaci Hüseyinoğlu et al. (2018), Huma et al. (2019), Knop (2019), Iqbal (2020), Chen et al. (2020), Weli et al. (2020)
Multiple data analysis method	Bienstock et al. (1997), Mentzer et al. (1999), Mentzer et al. (2001), Stank et al. (2003), Rabinovich and Bailey (2004), Panayides and So (2005), Feng et al. (2007), Gil-Saura et al. (2008), Bienstock et al. (2008), Birdogan et al. (2009), Juga et al. (2010), Gil-Saura et al. (2010), Fugate et al. (2010), Kersten and Koch (2010), Rao et al. (2011), Thai (2013), Gil-Saura and Ruiz-Molina (2011), Kilibarda et al. (2012), Jang et al. (2013), Giovanis et al. (2013), Jang et al. (2014), Philipp and Grant (2015), Vural and Tuna (2015), Zailani et al. (2018), Le et al. (2019), Vazifehdan and Darestani (2019), Xu et al. (2019), Vu et al. (2019), Hong and Nguyen (2020)

Table 4 provides an overview of the ratio of used LSQ measurement models and, it is clear that most of the researchers preferred to create a unique service quality measurement model for logistics or preferred to add a modification to generally used methods instead of directly using developed and proved reliable methods. Logistics services have been always a chain of multiple services and findings may show differences among supply field, region or service expertise. For instance, Zailani et al. (2018) focused on LSQ considering halal logistics network and developed an individual service quality model. Thai (2008) has provided a service quality method for port operations and defined six brand new dimensions: resources, outcome, process, management, image or reputation and social responsibility. Despite having specialized service quality measurement models for logistics operations, most of the researchers have used the classical model of SERVQUAL in quantitative research. This approach also provides an insight into the inefficient LSQ measurement model for general usage.

**Table 4:** Service Quality Measurement Models in Selected Publications

LSQ measurement model	Publication ratio (%)
SERVQUAL	49
SERVPERF	2
LSQ Scale	32
Statistical Scale (EFA, CFA, ANOVA, SEM, MANOVA)	15
Others	2

In addition, the LSQ scale created by Mentzer et al. (1999, 2001) has been used by researchers particularly. Rafiq and Jaafar (2007) had used the LSQ scale to measure customer perception on 3PL service providers, authors suggested generalizability of the LSQ scale on a similar sample model. Bouzaabia et al. (2013) has utilized the LSQ scale to compare the LSQ perception between Romania and Tunisia in retail logistics. Yumurtaci Huseyinoglu et al. (2018) has investigated the service quality scale model on Omni-channel capability. Table 5 provides an overview of LSQ dimensions and how often they are used in literature. The publication list has been submitted in chronological order to provide an overview of the development of LSQ dimensions that have been used throughout the period of the systematic research analysis. Due to different naming conventions on similar meanings, LSQ dimensions have been grouped by their relevance to each other. As a result, most frequently used LSQ measurement dimensions have been identified. Dimensions related to communication have been used 27 times in total, which have been mentioned under different names such as personal contact quality; responsiveness; customer focus etc. Second most frequently used LSQ dimensions are process-related and have been mentioned 20 times in the selected sample publications. Process related dimensions have been mentioned as order release quantities, order accuracy, order discrepancy handling, order quality and correctness, etc. Third but not least used dimensions are time-related and have been used 19 times in publications throughout the period. Time-related dimensions have been named in different forms such as timeliness, on-time delivery, lead time, etc. Over time, it has become clearly visible that while the focus of the operational quality has lost its importance and significance, communication-related dimensions and empathy dimension usage and their relation to quality have gained importance due to factors such as, responsiveness, empathy, personnel contact quality, etc.

**Table 5:** Service Quality Dimensions Among the Selected Sample

LSQ dimensions	Publication	Frequency
Time Related Dimensions (timeliness, on-time delivery, order processing time, lead-time, etc.)	Bienstock et al. (1997), Mentzer et al. (1999), Franceschini and Rafele (2000), Mentzer et al. (2001), Panayides and So (2005), Rafiq and Jaafar (2007), Feng et al. (2007), Gil-Saura et al. (2008), Bienstock et al. (2008), Gotzamani et al. (2010), Kilibarda et al. (2012), Thai (2013), Bouzaabia et al. (2013), Politis et al. (2014), Philipp and Grant (2015), Yu et al. (2016), Murfield et al. (2017), Zailani et al. (2018), Yumurtaci Hüseyinoğlu et al. (2018)	19
Process Related Dimensions (order release quantities, order accuracy, order discrepancy handling, order quality and correctness, etc.)	Mentzer et al. (1997), Bienstock et al. (1997), Durvasula et al. (1999), Franceschini and Rafele (2000), Mentzer et al. (2001), Panayides and So (2005), Rafiq and Jaafar (2007), Feng et al. (2007), Gil-Saura et al. (2008), Thai (2008), Bienstock et al. (2008), Gotzamani et al. (2010), Kilibarda et al. (2012), Thai (2013), Bouzaabia et al. (2013), Politis et al. (2014), Philipp and Grant (2015), Yu et al. (2016), Zailani et al. (2018), Yumurtaci Hüseyinoğlu et al. (2018)	20
Communication Related Dimensions (personal contact quality, responsiveness, customer focus, etc.)	Fung and Wong (1998), Mentzer et al. (1999), Durvasula et al. (1999), Franceschini and Rafele (2000), Mentzer et al. (2001), Rafele (2004), Panayides and So (2005), Seth et al. (2006), Davis and Mentzer (2006), Rafiq and Jaafar (2007), Feng et al. (2007), Birdogan et al. (2009), Thai (2013), Bouzaabia et al. (2013), Politis et al. (2014), Roslan et al. (2015), Philipp and Grant (2015), Vural and Tuna (2015), Kilibarda et al. (2016), Limbourg et al. (2016), Sohn et al. (2017), Zailani et al. (2018), Yumurtaci Hüseyinoğlu et al. (2018), Knop (2019), Le et al. (2019), Chen et al. (2020), Iqbal (2020)	27
Tangibility	Durvasula et al. (1999), Rafele (2004), Seth et al. (2006), Davis and Mentzer (2006), Birdogan et al. (2009), Kilibarda et al. (2016), Limbourg et al. (2016), Sohn et al. (2017), Knop (2019), Le et al. (2019), Chen et al. (2020)	11
Operational and Relational Quality	Stank et al. (2003), Juga et al. (2010), Jang et al. (2013), Huma et al. (2019), Weli et al. (2020)	5
Empathy	Durvasula et al. (1999), Panayides and So (2005), Seth et al. (2006), Davis and Mentzer (2006), Birdogan et al. (2009), Roslan et al. (2015), Kilibarda et al. (2016), Limbourg et al. (2016), Sohn et al. (2017), Knop (2019), Le et al. (2019), Chen et al. (2020)	12
Reliability	Durvasula et al. (1999), Panayides and So (2005), Seth et al. (2006), Davis and Mentzer (2006), Kilibarda et al. (2012), Roslan et al. (2015), Kilibarda et al. (2016), Limbourg et al. (2016), Sohn et al. (2017), Knop (2019), Le et al. (2019)	11
Image and Social Responsibility	Thai (2008), Thai (2013), Vural and Tuna (2015), Lan et al. (2016)	4

The findings have indicated that the LSQ research area has remained incomplete in the literature. Thus, tailored service quality with hierarchical dimensions for logistics services are more applicable to analyze LSQ. Dimensions have been selected based on their relevance and frequency of use. As it has been noticed from the studies, focus on customer-related services in logistics operations is increasing, therefore, dimensions related to customer focus quality have been selected as the first dimension for this study to analyze further in the interviews. Additionally, by the image of the company and social responsibility acts investigated under a total of six LSQ dimensions and twenty sub-factors have been identified by their relevancy on logistics and the frequency of the use: Information quality, customer focus quality, order fulfillment quality, timeliness quality, corporate image and social responsibility were selected.

## Semi-structured interviews

Semi-structured interviews allow participants and the interviewer to interchange knowledge within mutual benefit and, allow the interviewer to ask follow-up questions to participants based on the development of the answers (Rubin and Rubin 2005). In order to benefit from the professional view of the participants, semi-structured interview method has been selected. Semi-structured interview method is considered more fit for further investigation on LSQ dimensions because the topic is broadly discussed and has no consensus has been reached either on the definition or on the quality dimensions. Semi-structured interview has allowed participants to roam freely around the topic, and follow-up questions have provided preferable inputs and modifications on developed LSQ dimensions and sub-factors. As shown in Table 6, interviews were carried out with five logistics business professionals. Two of the participants were logistics managers in retail business, one was the logistics service provider team lead and two of them were logistics specialists for logistics service providers. All interview participants and their companies were located in Germany and companies have the scope of working in global logistics and supply chain businesses. All interviews have been conducted through online calls, and meetings have been recorded. Five interviews lasted average of thirty minutes for each participant.

**Table 6:** Semi-structured Interview Table

Role of the participant	Experience (years)	Type of organization	Business area	Interview length (min)	Method
Senior manager	11	3PL service provider	Logistics	25	Online meeting
Logistics specialist	5	3PL service provider	Logistics	20	Online meeting
Logistics specialist	5	3PL service provider	Logistics	25	Online meeting
Manager	7	LSP’s customer	Retail	30	Online meeting
Manager	6	LSP’s customer	Retail	35	Online meeting

Semi-structured interview questions have been designed according to the outcome of the systematic literature review. Open-ended questions have invited participants to follow-up the topic. Open-ended questions have been designed for each participant and their companies. Next set of questions have been designed for each quality dimension that has been identified in the systematic literature analysis and the said questions asked participants their point of view to validate and modify the proposed model. In general, participants have been directed with general questions to understand their personal quality perceptions and followed-up with prompt questions.

As a result, construction of the preliminary proposed quality dimensions has changed. All participants have expressed the importance of their customer value and its relation with quality perception, also they have highlighted that quality dimension is in fact a customer obsession. Therefore, naming has been changed to ‘*customer obsession quality’* from ‘*customer focus quality’*. Additionally, all participants have highlighted and agreed on the social responsibility activities are related to companies’ image; therefore, LSQ dimensions have merged under one quality dimension: social responsibility and company image. Additionally, LSQ factors have also been discussed and modified as a consequence of the interviews. Sub-dimensional quality factors have raised to 24 from 20 in total. Final LSQ dimensions and factors have been defined as shown in Table 7.

**Table 7:** LSQ Dimensions and Factors

Customer Obsession Quality	Employees’ approach and behavior while meeting customers’ requests
	Responsiveness of employees to customers’ needs
	Competency of employees to customers’ questions and order needs
	Employees’ knowledge on customers’ needs and requests
	Handling customer feedback
Order Fulfilment Quality	Order accuracy
	Order condition
	Order discrepancy handling
	Consistency of service performance
	Safety and security in delivery
	Reliability, regularity, flexibility and availability of service
Timeliness Quality	Total order cycle time
	Transportation time
	Back-order time
	Timeliness of shipment pickup and delivery
	Order placement accessibility and handiness
Information Quality	Application of IT and EDI in customer service
	Innovative solutions in logistics services
	Availability of order information
	Shipment tracing capability
Corporate Image and Social Responsibility	Company’s ethical image
	Social responsibility in management and concerning human safety in operations
	Environmentally friendly operations
	Company’s reputation for reliability

After the final evaluation of semi-structured reviews, shipment tracing capability, innovative solutions in logistics services; reliability, regularity, flexibility and availability of service, company’s reputation for reliability have been added to the LSQ factors and LSQ scale has been developed with five quality dimensions and 24 factors in total.

## Research findings

Research findings have been developed with qualitative research techniques. Firstly, systematic literature analysis has been applied to the LSQ related papers with specified criteria between 1995 and 2020. Samples have been analyzed with systematically created filtering and descriptive analysis. Results have been analyzed and shown that researchers have not reached a consensus either on the LSQ perception or the measurement method. Additionally, a paradigm shift towards customer-oriented services from the natural physical movement of the cargoes has been observed in recent years. As a result, logistics service customers are giving more importance to business-to-business or business-to-customer communication and empathy. This change has been seen in the recent LSQ publications as well. As a consequence of the initial analysis, six dimensions and twenty logistics factors have been developed. Preliminary findings have been discussed in five semi-structured interviews. Logistics professionals’ contributions have been included in this study to ensure that literature key findings are in line with actual business and quality dimensions have improved by the outcome of the results.

As a result, systematic literature analysis has shown that SERVQUAL quality measurement method is still broadly used; however, there have been great contributions from many authors towards LSQ and the creation of logistics specific quality measurement model. Despite these improvements, there has been no consensus on the singular quality measurement model. This research proposes LSQ dimensions and factors created from systematic literature analysis and semi-structured interviews. Firstly, six-dimensional twenty factors have been developed and findings have been improved after the semi-structured interviews. Final model proposes five LSQ dimensions with twenty-four factors.

## Conclusion and recommendations for further researches

Logistics services have been continuously growing around the world. These improvements and developments have increased the competition among service providers. There has been an increment in the number of research papers exploring this area. Service providers are trying to leverage operational excellence with high quality of services to maintain customer satisfaction, loyalty, and market competition. A regularly dynamic environment requires dynamic solutions, therefore, logistics services are constantly in development. Consequently, the perception of LSQ has been changing.

It has been found that LSQ understanding and applications have been evolving around the business focus of LSPs. Throughout the development of the quality dimensions in logistics, there have been different approaches from different authors. In the literature, the focus of the LSQ dimensions has been differing among different periods of the samples and it clearly shows the change in the focus on the quality. After observing a period of twenty years, early developed LSQ dimensions have shown that quality focus is mainly on the physical attributes of the operations, such as physical distribution and timeliness related dimensions. Over time, logistics services have accumulated more customer-oriented operations hence, in later periods customer-related LSQ dimensions have been observed, such as personal service/contact, empathy. The dimensional switch has also been accepted in semi-structured interviews and recorded as the most important dimension of the LSPs. Therefore, currently keeping positive relations with customers by providing emphatic continuous relationship has been more important for LSPs.

Despite having a high rate in empirical studies, findings suggest that researchers used repeatedly SERVQUAL model in LSQ measurement even though there have been measurement models created specifically for logistics services. This indicates that the search of the LSQ dimensions and measurement methods have not been completed; hence, it is open for improvement and eventually reaching the recognized LSQ measurement method. This study is providing a framework for service quality in logistics for researchers and logistics professionals by systematically analyzing the previously developed studies and measurement models. Primary quality dimensions have been developed from systematic literature analysis by systemizing and organizing the existing literature. Then, additional interviews have been conducted with service professionals. As a result, framework of LSQ has been developed with five dimensions with customer obsession quality, order fulfillment quality, timeliness quality, information quality, corporate image and social responsibility and twenty-four factors. The holistic approach of the research model has asserted LSQ dimensions for further measurement models.

Proposed model may be used as a framework for further studies and can be strengthened by empirically testing in multiple regions of the world. LSQ dimensions may be improved by conducting focus group meetings and additional interviews with logistics professionals from different regions of the world. Additionally, professionals may use these LSQ dimensions as an internal quality indicator and use factors and dimensions as quality key performance metrics. Managers may benefit from the findings to create quality-oriented logistics services or improve existing service models.

## Data Availability

The datasets used and analyzed during the current study are available from the corresponding author on reasonable request.

## Abbreviations

LSQ: Logistics service quality
LSP: Logistics Services providers
SERQUAL: Service quality
SERVPERF: Service performance
